# Supplementary figures and images for: Dissection of MAPK signaling specificity through protein engineering in a developmental context
Source: BMC Plant Biol. 2018 Apr 10;18:60. doi: 10.1186/s12870-018-1274-9 (PMC5894206; doi:10.1186/s12870-018-1274-9)

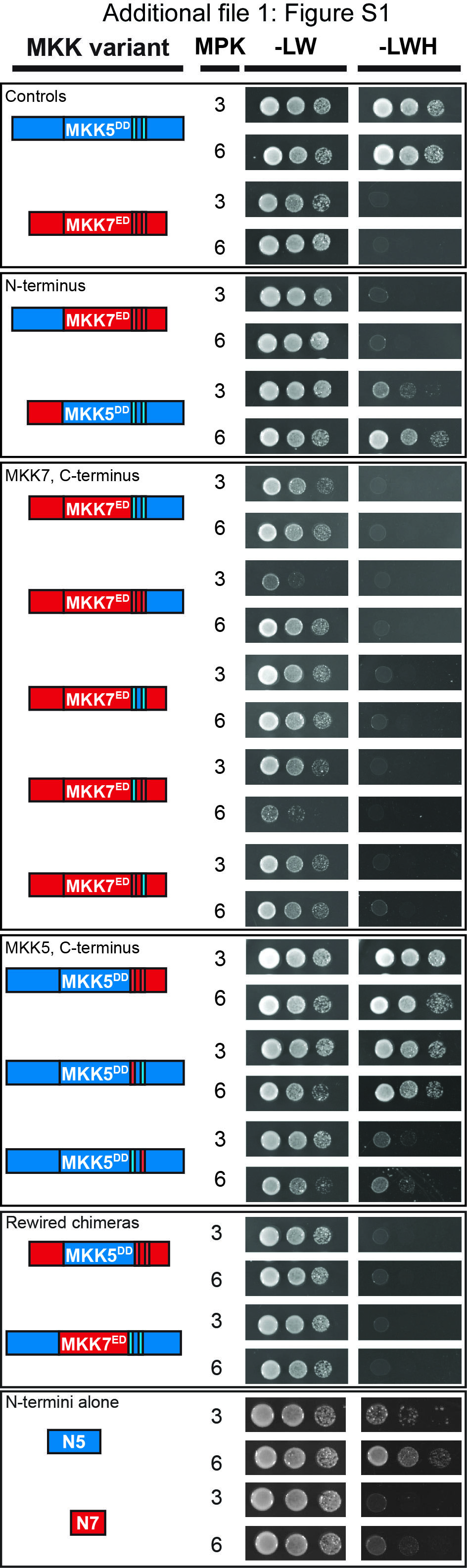

Supplement: Supplementary file 1 — Figure S1. Yeast two-hybrid assay with native and chimeric MKKs and MPK3/MPK6. Representative yeast two-hybrid assay between MKK chimeras and MPK3/6 at 3 days of growth on control (-LW) and interaction (-LWH); three patches are serial (10 fold) dilutions. (JPEG 607 kb) [file 12870_2018_1274_MOESM1_ESM.jpg]

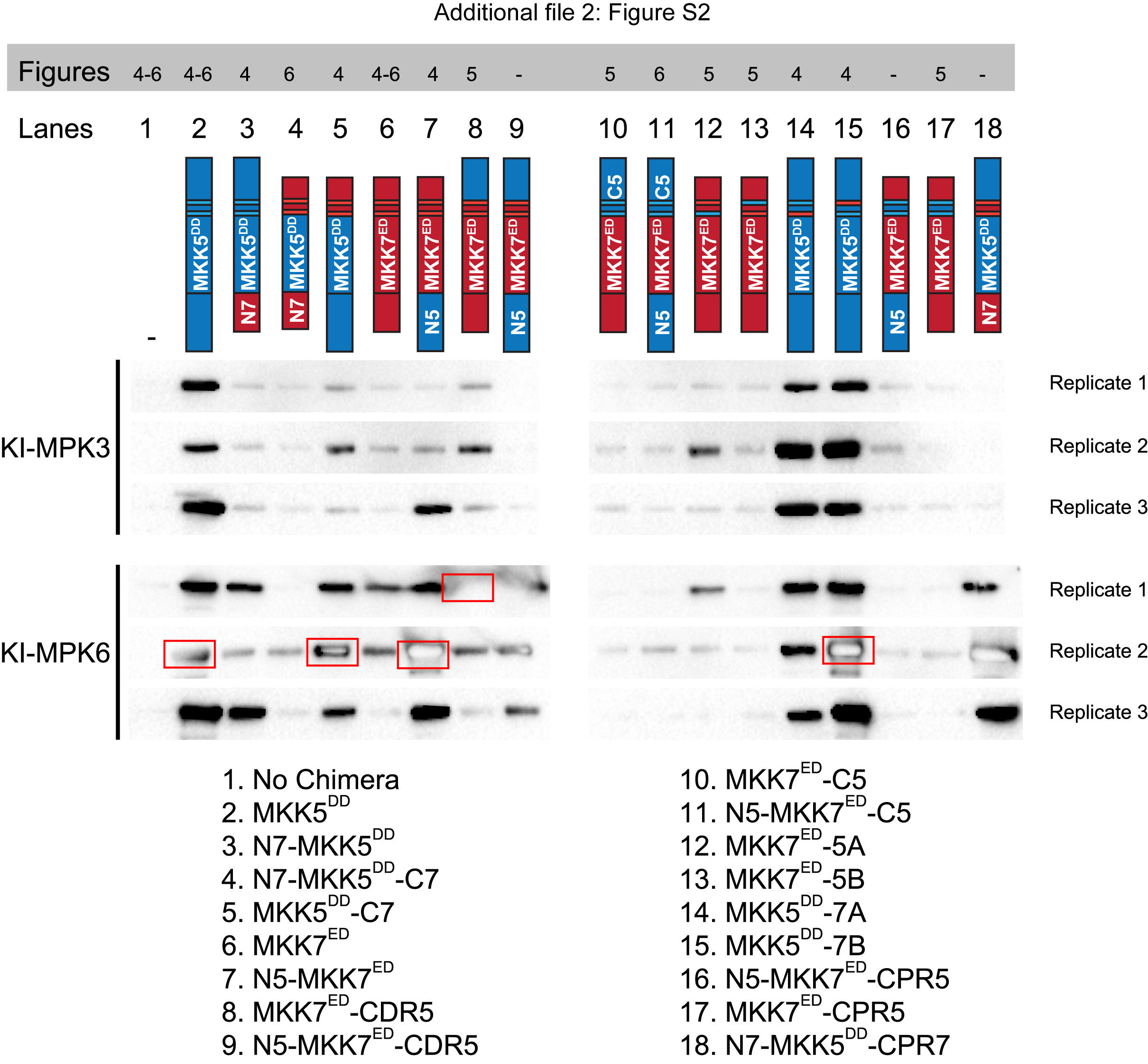

Supplement: Supplementary file 2 — Figure S2. In vitro kinase assays of MKKs and chimeras using kinase inactive (KI) MPK3 and MPK6 as substrates. Phosphorylation assays were performed as described in methods. Samples were separated in SDS-PAGE, transferred to PVDF membranes and probed with anti-pERK antibody. Western blots were quantified and average of experiments was used as an estimation of in vitro activity. Each lane is labeled with the figure where the data is presented (grey bar marked Figures), the kinase used with a graphic representation and with its full name in bottom panel. Samples unassigned to any figure (labeled as “-” in Figure) were not presented in the main figures of this manuscript for the sake of brevity, but are included here to allow us to keep the blots intact. Red squares highlight samples that were not considered for quantification due to detection artifacts. (JPEG 565 kb) [file 12870_2018_1274_MOESM2_ESM.jpg]

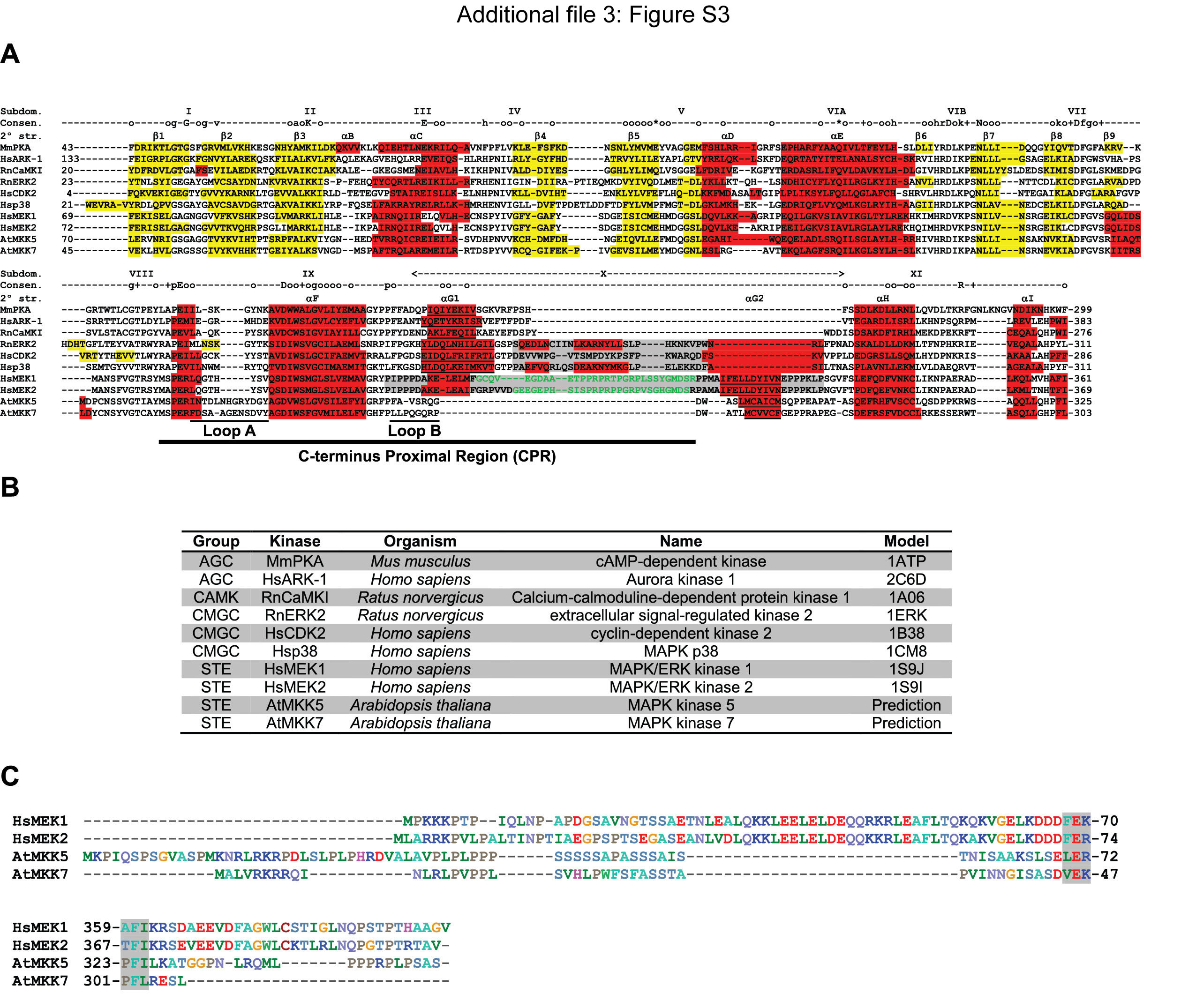

Supplement: Supplementary file 3 — Figure S3. Multiple sequence alignment [43] of catalytic domains in MPK kinases from mouse, rat, human and Arabidopsis thaliana. A, Conserved subdomains (Subdom.) and consensus sequences (Consen.) are represented on top of the alignment and follow the same codes and convention for an alignment of 60 different kinases by Hanks and Hunter [28]. In the consensus line: uppercase letters, invariant residues; lowercase residues, nearly invariant residues; o, positions conserving nonpolar residues; *, positions conserving polar residues; +, positions conserving small residues with near neutral polarity. Mammalian representative kinases selected for the alignment have been crystalized and belong to AGC group [cAMP-dependent kinase, cGMP-dependent kinase, etc.], CAMK group [Calcium-calmoduline-dependent protein kinase], CMGC group [cyclin-dependent kinase, mitogen-activated kinase, glycogen synthase kinase and cyclin-dependent-like kinase] and STE group [homologues of STE11 and STE20]. Gray boxes show CMGC insert [30] in ERK2, CDK2 and p38, and Pro-rich sequence (PRS, involved in binding the scaffold MP1) in MEK1 and MEK2 were included in the alignment and cause an expansion of subdomain X. Secondary structure (2° str.) information is overlaid in the alignment (red for α-helices and yellow for β-strands). Conserved α-helices and β-strands are labeled following convention [28, 31]. Due to CMGC insert and PRS, αG helix is located in two different regions of the alignment and was named differently (residues underlined): αG1 for MmPKA, HsARK-1, RnCaMKI, RnERK2, HsCDK2 and Hsp38; and αG2 for HsMEK1/2 and AtMKK5/7. In green text, missing residues in crystal structures from HsMEK1 and HsMEK2 which include the PRS. B, Table provides general information and structural model names for kinases used in this comparison. C, Multiple sequence alignment of N- and C-termini from human MEK1/2 and Arabidopsis MKK5/7. Sequences highlighted in gray correspond to first and last three amino acids [file 12870_2018_1274_MOESM3_ESM.jpg]

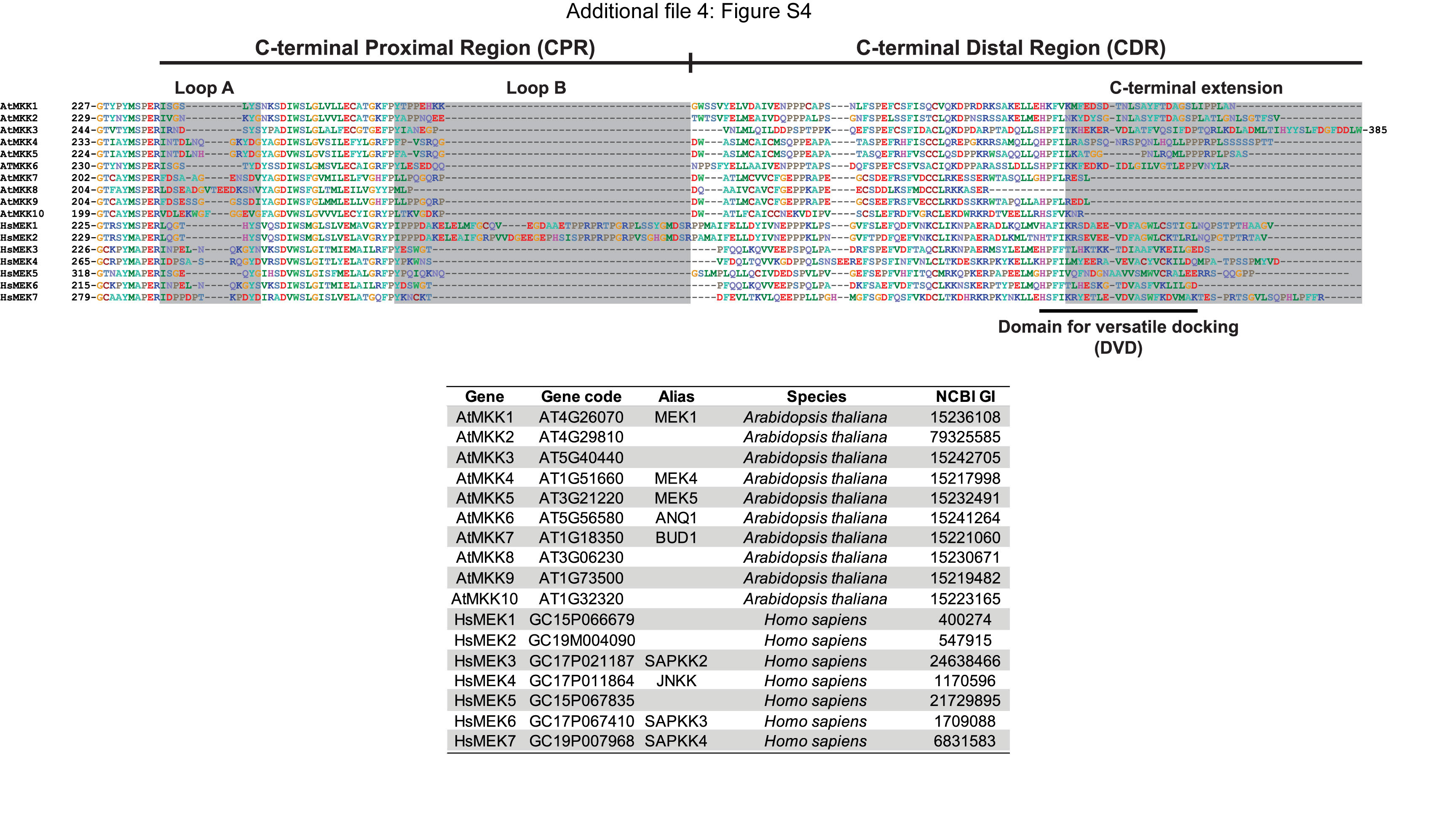

Supplement: Supplementary file 4 — Figure S4. Multiple sequence alignment of C-termini in Arabidopsis and human MKKs. Top panel, partial sequences for all Arabidopsis and human MKKs were aligned with ClustalOmega. Loops A and B defined for Arabidopsis MKKs are highlighted in gray. Number of first amino acid of the partial sequence is noted to the left of each sequence. The long C-terminal extension of AtMKK3 is truncated in this figure. Bottom panel, genes used in the alignment. Name of genes for Arabidopsis correspond to Arabidopsis Genome Initiative (AGI) codes and GeneCards (GC) for humans. NCBI GI, National Center for Biotechnology Information protein sequence identifier. (JPEG 1010 kb) [file 12870_2018_1274_MOESM4_ESM.jpg]

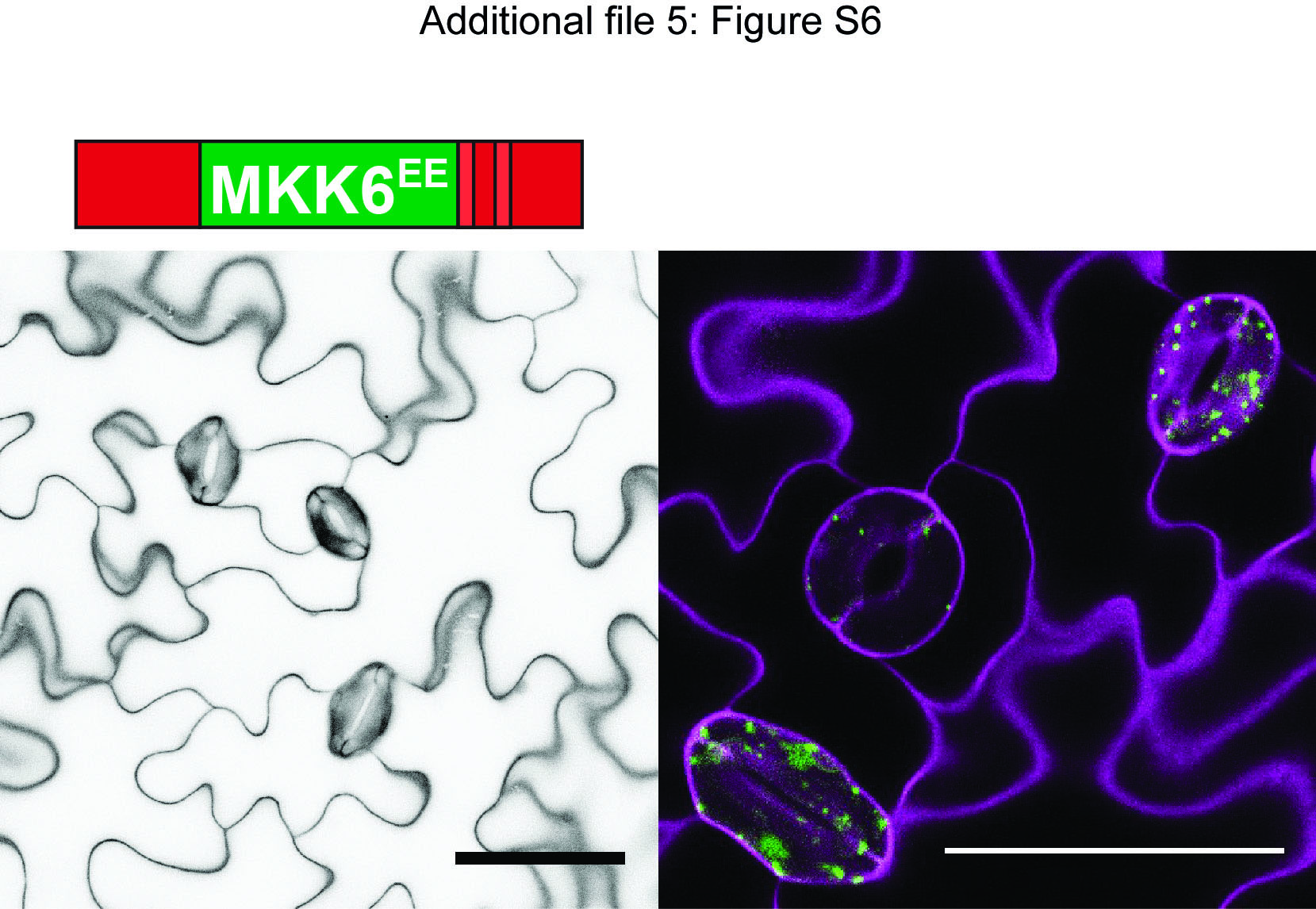

Supplement: Supplementary file 5 — Figure S6. Representative phenotype and subcellular localization of transgenic seedlings expressing FAMAp:N7-MKK6EE-C7. (JPEG 465 kb) [file 12870_2018_1274_MOESM5_ESM.jpg]

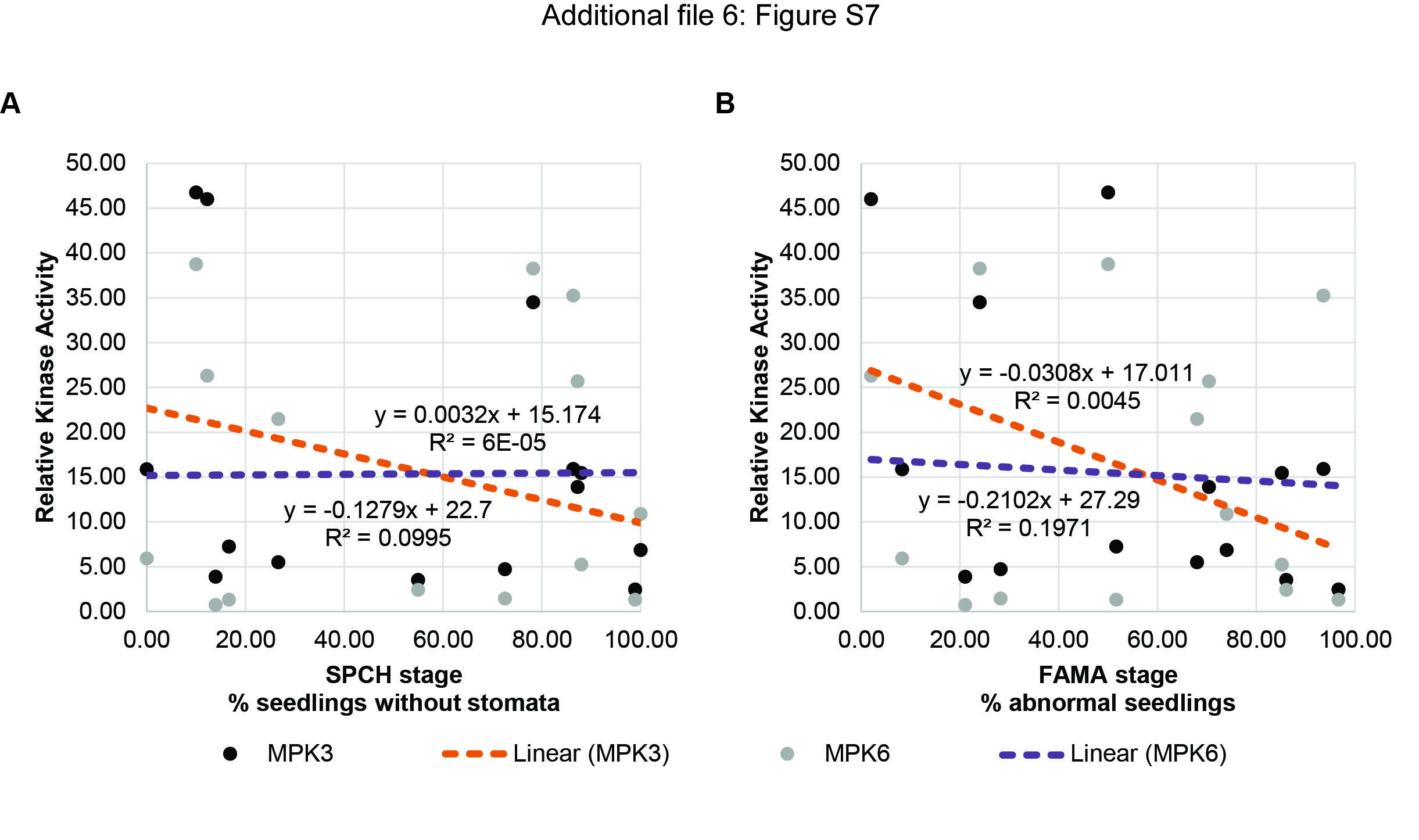

Supplement: Supplementary file 6 — Figure S7. In vitro kinase activity of MKKs does not correlate with their in vivo activity. Linear regressions for MPK3 or MPK6 activity versus SPCH stage (A) or FAMA stage (B) activities, with their formulas and R2, are displayed in the figure. FAMA stage activity was calculated as the addition of phenotypes different than normal (Inhibited, Small and Large clusters in Table 1). (JPEG 505 kb) [file 12870_2018_1274_MOESM6_ESM.jpg]

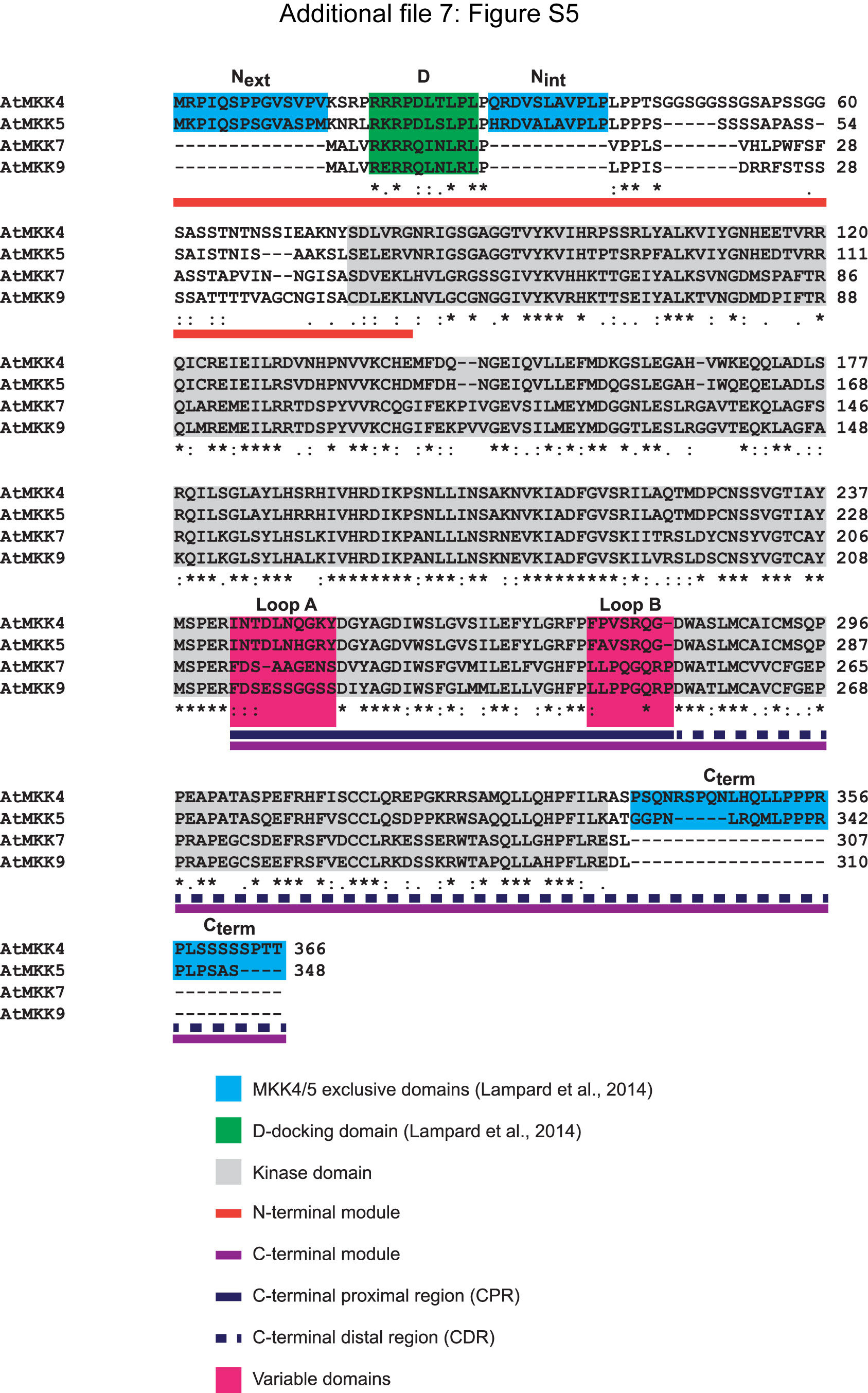

Supplement: Supplementary file 7 — Figure S5. Multiple sequence alignment for MKK4, MKK5, MKK7 and MKK9. Domains described in [23] are highlighted in blue and green. (JPEG 779 kb) [file 12870_2018_1274_MOESM7_ESM.jpg]
