# Supplementary material for: Dissection of MAPK signaling specificity through protein engineering in a developmental context
Source: BMC Plant Biol. 2018 Apr 10;18:60. doi: 10.1186/s12870-018-1274-9 (PMC5894206; doi:10.1186/s12870-018-1274-9)
Supplement: Supplementary file 8 — Oligonucleotides sequences used in this paper, cloning strategy and protein sequence of chimeras. (DOCX 39 kb) [file 12870_2018_1274_MOESM8_ESM.docx]

Title

**Dissection of MAPK signaling specificity through protein engineering in a developmental context**

Diego L. Wengier, Gregory R. Lampard and Dominique C. Bergmann

**Additional file 8**

**Construction of chimeras**

Domain swap constructs were assembled by fusion PCR from DNA amplicons (blocks) generated with Phusion® High-Fidelity DNA Polymerase following manufacturer’s instructions (New England Biolabs, Ipswich, MA). To generate blocks, MKK5^DD^ and MKK7^ED^ cloned into pENTR without stop codons or other chimeras were used as templates ([Lampard et al, 2009](#_ENREF_22)). Blocks were designed to contain attL1 and attL2 functional sequences from pENTR to ease the cloning procedure through the Gateway strategy (Table S1, A and B). For domain swaps assembled from two blocks, 5’ blocks contained the M13 forward priming site and attL1 recombination site before the MKK sequence; and 3’ blocks contained the MKK sequence followed by attL2 recombination site and M13 reverse priming site. To facilitate fusion of the blocks, reverse primers for 5’ blocks and forward primers for 3’ blocks were designed as chimeras of the two blocks to be fused, containing at least 15 bases from each block, and were completely complementary to each other. PCR products were gel extracted using QIAquick Gel Extraction Kit (QIAGEN Inc., Valencia, CA) and 1:1 molar ratio mix were used as templates on fusion PCR reactions using M13 forward and reverse primers. Domain swap constructs were gel purified and cloned into pJET 1.2 according to CloneJET PCR Cloning Kit instructions (Thermo Life Sciences, Pittsburgh, PA). For domain swap constructs assembled from 3 blocks, 5’ and 3’ were generated with the same strategy as above, while internal block was amplified with forward and reverse chimeric primers. As domain swaps became more elaborate, first domain swap constructs were used as templates for generating new blocks. Primers, templates and sequences for each domain swap are listed in Table S1, A and B.

To build constructs for expression under SPCH and FAMA promoters, 2.5-kb fragments previously described ([MacAlister et al, 2007](#_ENREF_26); [Ohashi-Ito & Bergmann, 2006](#_ENREF_33)) were first adapted to the Multisite Gateway system. Promoters were shuttled from pENTR to pDONR P4 P1R (Life Technologies, Grand Island, NY) by PCR amplification using promoter shuttling primers (Table S1A) followed by BP recombination performed under manufacturer’s instructions. Promoters flanked by attL4 and attR1 recombination sites (pDONR-promoter) were used in Multisite recombination reactions with domain swap constructs in pJET and R4pGWB440 [destination vector carrying the Gateway cassette flanked by attR4 and attR2 recombination sites, in frame C-terminal fusion to enhanced YFP and kanamycin selection in plants ([Nakagawa et al, 2008](#_ENREF_32))]. Recombination reactions were performed in a two-step protocol. First, 1 µl of LR Clonase II was added to 4 µl vector mix (containing 150 ng of pDONR-promoter and 150 ng pJET-domain swap construct) and incubated at 25ºC for 5 hours. Then, 150 ng of R4pGWB440 in 4 µl solution were added to the reactions along 1 µl of LR Clonase II. Reactions were incubated for additional 16 hours at 25ºC and then stopped after the addition of 1 µl of Proteinase K and incubation for 10 min at 37ºC. Constructs were confirmed by sequencing and introduced in *Arabidopsis thaliana* var. *Col-0* by *Agrobacterium tumefaciens*-mediated transformation.

For *mpk6-3* transformation, FAMAp:N5-MKK7^ED^ construct was PCR amplified with FAMAp F and MKK7 No Stop R primers, introduced into pENTR/D-TOPO and recombined to pHGY binary vector (Hygromycin B resistance in plants).

**Table S1: Oligonucleotides used in this paper (A) and combination of PCR fragments to generate chimeras (B).**

A.

| PCR # | Name | Sequence | Second primer | Template | Amplicon |
| --- | --- | --- | --- | --- | --- |
|  | M13F | GTAAAACGACGGCCAG |  |  |  |
|  | M13R | GTCATAGCTGTTTCCTG |  |  |  |
| 1 | N7-5-F | GATGTCGAGAAACTCAACCGTATCGGAAGC | M13R | pENTR-MKK5^DD^ | (N7)-MKK5 |
| 2 | N7-5-F |  | 5-C7-R | pENTR-MKK5^DD^ | (N7)-MKK5-(C7) |
| 3 | N7-5-R | GCTTCCGATACGGTTGAGTTTCTCGACATC | M13F | pENTR-MKK7^ED^ | N7-(MKK5) |
| 4 | N5-7-F | GAGCTAGAACGAGTGCACGTTCTCGGAAGA | M13R | pENTR-MKK5^DD^ | (N5)-MKK7 |
| 5 | N5-7-F |  | 7-C5-R | pENTR-MKK7^ED^ | (N5)-MKK7-(C5) |
| 6 | N5-7-R | TCTTCCGAGAACGTGCACTCGTTCTAGCTC | M13F | pENTR-MKK7^ED^ | N5-(MKK7) |
| 7 | 7-C5-F | ATGAGCCCGGAGAGAATTAACACTGATTTG | M13R | pENTR-MKK5^DD^ | (MKK7)-C5 |
| 8 | 7-C5-R | CAAATCAGTGTTAATTCTCTCCGGGCTCAT | M13F | pENTR-MKK7^ED^ | MKK7-(C5) |
| 9 | 5-C7-F | ATGAGTCCTGAGAGGTTTGACTCTGCCGCC | M13R | pENTR-MKK5^DD^ | (MKK5)-C7 |
| 10 | 5-C7-R | GGCGGCAGAGTCAAACCTCTCAGGACTCAT | M13F | pENTR-MKK7^ED^ | MKK5-(C7) |
| 11 | CP5-7 F | GTGAGTAGACAAGGTGACTGGGCGACGTTA | M13R | pENTR-MKK7^ED^ | (CP5)-CD7 |
| 12 | CP5-7 R | TAACGTCGCCCAGTCACCTTGTCTACTCAC | M13F | pJET-MKK7^ED^-C5 | MKK7-CP5-(CD7) |
| 13 | CP7-5 F | CAGGGACAGAGACCTGATTGGGCTAGTCTT | M13R | pENTR-MKK5^DD^ | (CP7)-CD5 |
| 14 | CP7-5 R | AAGACTAGCCCAATCAGGTCTCTGTCCCTG | M13F | pJET-N7-MKK5^DD^-C7 | N7-MKK5-CP7-(CD5) |
| 15 | CP7-5 R |  | M13F | pENTR-MKK7^ED^ | MKK7-(CD5) |
| 16 | 5-CP7A-5F | GAAAACTCCGATGTTTATGCTGGAGATGTT | M13R | pENTR-MKK5^DD^ | (CP7A)-CP5B-CD5 |
| 17 | 5-CP7A-5R | AACATCTCCAGCATAAACATCGGAGTTTTC | M13F | pJET-MKK5^DD^-C7 | MKK5-CP7A-(CP5B) |
| 18 | 5-CP7B-5F | TTGGGGAGGTTTCCTTTGCTTCCTCAGGGA | M13R | pJET-N7-MKK5^DD^-CP7 | (CP5A)-CP7B-CD5 |
| 19 | 5-CP7B-5R | TCCCTGAGGAAGCAAAGGAAACCTCCCCAA | M13F | pENTR-MKK5^DD^ | MKK5-CP5A-(CP7B) |
| 20 | 7-CP5A-7F | GGTCGTTACGATGGTTACGCAGGCGATATC | M13R | pENTR-MKK7^ED^ | (CP5A)-CP7B-CD7 |
| 21 | 7-CP5A-7R | GATATCGCCTGCGTAACCATCGTAACGACC | M13F | pJET-MKK7^ED^-CD5 | MKK7-CP5A-(CP7B) |
| 22 | 7-CP5B-7F | GTCGGACATTTTCCGTTTGCTGTGAGTAGA | M13R | pJET-MKK7^ED^-CP5 | (CP7A)-CP5B-CD7 |
| 23 | 7-CP5B-7R | TCTACTCACAGCAAACGGAAAATGTCCGAC | M13F | pENTR-MKK7^ED^ | MKK7-CP7A-(CP5B) |
| 24 | N7-6-F | GATGTCGAGAAACTCAAAGTCATCGGAAAAGGC | 6-C7-R | pENTR-MKK6^EE^ | (N7)-MKK6-(C7) |
| 25 | N7-6-R | GCCTTTTCCGATGACTTTGAGTTTCTCGACATC | M13F | pENTR-MKK7^ED^ | N7-(MKK6) |
| 26 | 6-C7-F | ATGTCGCCTGAGAGGTTTGACTCTGCCGCC | M13R | pENTR-MKK7^ED^ | (MKK6)-C7 |
|  | 6-C7-R | GGCGGCAGAGTCAAACCTCTCAGGCGACAT |  |  |  |
| 24 | N5-F | CACCATGAAACCGATTCAATCT | N5-R | pENTR-MKK5^DD^ | N5 |
|  | N5-R | GTTCACTCGTTCTAGCTC |  |  |  |
| 25 | N7-F | CACCATGGCTCTTGTTCGTAAA | N7-R | pENTR-MKK7^ED^ | N7 |
|  | N7-R | GAGTTTCTCGACATCGGA |  |  |  |
| 26 | attB4F | GGGG ACA ACT TTG TAT AGA AAA GTT GAA AAA GCA GGC TCC GCG | attB1R | pENTR-PROMOTER | attB4-PROMOTER-attB1 |
|  | attB1R | GGGG AC TGC TTT TTT GTA CAA ACT TG AGC TGG GTC GGC |  |  |  |
| 27 | FAMAp F | CACCATCACTAAGTGGTTTTACTAGTG | MKK7 No Stop R | R4pGWB440-FAMAp:N5-MKK7^ED^ | FAMAp:N5-MKK7^ED^ |
|  | MKK7 No Stop R | AAGACTTTCACGGAGAAAAGGGTG |  |  |  |

B.

| PCR combination | Amplicon combination | Construct obtained |
| --- | --- | --- |
| 4 + 6 | (N5)-MKK7 + N5-(MKK7) | pJET-N5-MKK7^ED^ |
| 1 + 3 | (N7)-MKK5 + N7-(MKK5) | pJET-N7-MKK5^DD^ |
| 7 + 8 | (MKK7)-C5 + MKK7-(C5) | pJET-MKK7^ED^-C5 |
| 13 + 15 | (CP7)-CD5 + MKK7-(CD5) | pJET-MKK7^ED^-CD5 |
| 11 + 12 | (CP5)-CD7 + MKK7-CP5-(CD7) | pJET-MKK7^ED^-CP5 |
| 20 + 21 | (CP5A)-CP7B-CD7 + MKK7-CP5A-(CP7B) | pJET-MKK7^ED^-5A |
| 22 + 23 | (CP7A)-CP5B-CD7 + MKK7-CP7A-(CP5B) | pJET-MKK7^ED^-5B |
| 9 + 10 | (MKK5)-C7 + MKK5-(C7) | pJET-MKK5^DD^-C7 |
| 16 + 17 | (CP7A)-CP5B-CD5 + MKK5-CP7A-(CP5B) | pJET-MKK5^DD^-7A |
| 18 + 19 | (CP5A)-CP7B-CD5 + MKK5-CP5A-(CP7B) | pJET-MKK5^DD^-7B |
| 13 + 14 | (CP7)-CD5 + N7-MKK5-CP7-(CD5) | pJET-N7-MKK5^DD^-CP7 |
| 2 + 6 + 9 | (N7)-MKK5-(C7) + N5-(MKK7) + (MKK5)-C7 | pJET-N7-MKK5^DD^-C7 |
| 3 + 5 + 7 | N7-(MKK5) + (N5)-MKK7-(C5) + (MKK7)-C5 | pJET-N5-MKK7^ED^-C5 |
| 24 + 25 +26 | (N7)-MKK6-(C7) + N7-(MKK6) + (MKK6)-C7 | pJET-N7-MKK6^EE^-C7 |

**Protein sequences of chimeras**

Phosphomimic mutations in activation loop (D/E) are highlighted in red.

**Full length MKK5^DD^ and MKK7^ED^:**

>MKK5

MKPIQSPSGVASPMKNRLRKRPDLSLPLPHRDVALAVPLPLPPPSSSSSAPASSSAISTNISAAKSLSELERVNRIGSGAGGTVYKVIHTPTSRPFALKVIYGNHEDTVRRQICREIEILRSVDHPNVVKCHDMFDHNGEIQVLLEFMDQGSLEGAHIWQEQELADLSRQILSGLAYLHRRHIVHRDIKPSNLLINSAKNVKIADFGVSRILAQDMDPCNDSVGTIAYMSPERINTDLNHGRYDGYAGDVWSLGVSILEFYLGRFPFAVSRQGDWASLMCAICMSQPPEAPATASQEFRHFVSCCLQSDPPKRWSAQQLLQHPFILKATGGPNLRQMLPPPRPLPSAS...(TRANSITION TO YFP)

>MKK7

MALVRKRRQINLRLPVPPLSVHLPWFSFASSTAPVINNGISASDVEKLHVLGRGSSGIVYKVHHKTTGEIYALKSVNGDMSPAFTRQLAREMEILRRTDSPYVVRCQGIFEKPIVGEVSILMEYMDGGNLESLRGAVTEKQLAGFSRQILKGLSYLHSLKIVHRDIKPANLLLNSRNEVKIADFGVSKIITRELDYCNDYVGTCAYMSPERFDSAAGENSDVYAGDIWSFGVMILELFVGHFPLLPQGQRPDWATLMCVVCFGEPPRAPEGCSDEFRSFVDCCLRKESSERWTASQLLGHPFLRESL...(TRANSITION TO YFP)

**Chimeras:**

>N5-MKK7^ED^

MKPIQSPSGVASPMKNRLRKRPDLSLPLPHRDVALAVPLPLPPPSSSSSAPASSSAISTNISAAKSLSELERVHVLGRGSSGIVYKVHHKTTGEIYALKSVNGDMSPAFTRQLAREMEILRRTDSPYVVRCQGIFEKPIVGEVSILMEYMDGGNLESLRGAVTEKQLAGFSRQILKGLSYLHSLKIVHRDIKPANLLLNSRNEVKIADFGVSKIITRELDYCNDYVGTCAYMSPERFDSAAGENSDVYAGDIWSFGVMILELFVGHFPLLPQGQRPDWATLMCVVCFGEPPRAPEGCSDEFRSFVDCCLRKESSERWTASQLLGHPFLRESL...(TRANSITION TO YFP)

>N7-MKK5^DD^

MALVRKRRQINLRLPVPPLSVHLPWFSFASSTAPVINNGISASDVEKLNRIGSGAGGTVYKVIHTPTSRPFALKVIYGNHEDTVRRQICREIEILRSVDHPNVVKCHDMFDHNGEIQVLLEFMDQGSLEGAHIWQEQELADLSRQILSGLAYLHRRHIVHRDIKPSNLLINSAKNVKIADFGVSRILAQDMDPCNDSVGTIAYMSPERINTDLNHGRYDGYAGDVWSLGVSILEFYLGRFPFAVSRQGDWASLMCAICMSQPPEAPATASQEFRHFVSCCLQSDPPKRWSAQQLLQHPFILKATGGPNLRQMLPPPRPLPSAS...(TRANSITION TO YFP)

>MKK7^ED^-C5

MALVRKRRQINLRLPVPPLSVHLPWFSFASSTAPVINNGISASDVEKLHVLGRGSSGIVYKVHHKTTGEIYALKSVNGDMSPAFTRQLAREMEILRRTDSPYVVRCQGIFEKPIVGEVSILMEYMDGGNLESLRGAVTEKQLAGFSRQILKGLSYLHSLKIVHRDIKPANLLLNSRNEVKIADFGVSKIITRELDYCNDYVGTCAYMSPERINTDLNHGRYDGYAGDVWSLGVSILEFYLGRFPFAVSRQGDWASLMCAICMSQPPEAPATASQEFRHFVSCCLQSDPPKRWSAQQLLQHPFILKATGGPNLRQMLPPPRPLPSAS...(TRANSITION TO YFP)

>MKK7^ED^-CDR5

MALVRKRRQINLRLPVPPLSVHLPWFSFASSTAPVINNGISASDVEKLHVLGRGSSGIVYKVHHKTTGEIYALKSVNGDMSPAFTRQLAREMEILRRTDSPYVVRCQGIFEKPIVGEVSILMEYMDGGNLESLRGAVTEKQLAGFSRQILKGLSYLHSLKIVHRDIKPANLLLNSRNEVKIADFGVSKIITRELDYCNDYVGTCAYMSPERFDSAAGENSDVYAGDIWSFGVMILELFVGHFPLLPQGQRPDWASLMCAICMSQPPEAPATASQEFRHFVSCCLQSDPPKRWSAQQLLQHPFILKATGGPNLRQMLPPPRPLPSAS...(TRANSITION TO YFP)

>MKK7^ED^-CPR5

MALVRKRRQINLRLPVPPLSVHLPWFSFASSTAPVINNGISASDVEKLHVLGRGSSGIVYKVHHKTTGEIYALKSVNGDMSPAFTRQLAREMEILRRTDSPYVVRCQGIFEKPIVGEVSILMEYMDGGNLESLRGAVTEKQLAGFSRQILKGLSYLHSLKIVHRDIKPANLLLNSRNEVKIADFGVSKIITRELDYCNDYVGTCAYMSPERINTDLNHGRYDGYAGDVWSLGVSILEFYLGRFPFAVSRQGDWATLMCVVCFGEPPRAPEGCSDEFRSFVDCCLRKESSERWTASQLLGHPFLRESL...(TRANSITION TO YFP)

>MKK7^ED^-5A

MALVRKRRQINLRLPVPPLSVHLPWFSFASSTAPVINNGISASDVEKLHVLGRGSSGIVYKVHHKTTGEIYALKSVNGDMSPAFTRQLAREMEILRRTDSPYVVRCQGIFEKPIVGEVSILMEYMDGGNLESLRGAVTEKQLAGFSRQILKGLSYLHSLKIVHRDIKPANLLLNSRNEVKIADFGVSKIITRELDYCNDYVGTCAYMSPERINTDLNHGRYDGYAGDIWSFGVMILELFVGHFPLLPQGQRPDWATLMCVVCFGEPPRAPEGCSDEFRSFVDCCLRKESSERWTASQLLGHPFLRESL...(TRANSITION TO YFP)

>MKK7^ED^-5B

MALVRKRRQINLRLPVPPLSVHLPWFSFASSTAPVINNGISASDVEKLHVLGRGSSGIVYKVHHKTTGEIYALKSVNGDMSPAFTRQLAREMEILRRTDSPYVVRCQGIFEKPIVGEVSILMEYMDGGNLESLRGAVTEKQLAGFSRQILKGLSYLHSLKIVHRDIKPANLLLNSRNEVKIADFGVSKIITRELDYCNDYVGTCAYMSPERFDSAAGENSDVYAGDIWSFGVMILELFVGHFPFAVSRQGDWATLMCVVCFGEPPRAPEGCSDEFRSFVDCCLRKESSERWTASQLLGHPFLRESL...(TRANSITION TO YFP)

>MKK5^DD^-C7

MKPIQSPSGVASPMKNRLRKRPDLSLPLPHRDVALAVPLPLPPPSSSSSAPASSSAISTNISAAKSLSELERVNRIGSGAGGTVYKVIHTPTSRPFALKVIYGNHEDTVRRQICREIEILRSVDHPNVVKCHDMFDHNGEIQVLLEFMDQGSLEGAHIWQEQELADLSRQILSGLAYLHRRHIVHRDIKPSNLLINSAKNVKIADFGVSRILAQDMDPCNDSVGTIAYMSPERFDSAAGENSDVYAGDIWSFGVMILELFVGHFPLLPQGQRPDWATLMCVVCFGEPPRAPEGCSDEFRSFVDCCLRKESSERWTASQLLGHPFLRESL...(TRANSITION TO YFP)

>MKK5^DD^-7A

MKPIQSPSGVASPMKNRLRKRPDLSLPLPHRDVALAVPLPLPPPSSSSSAPASSSAISTNISAAKSLSELERVNRIGSGAGGTVYKVIHTPTSRPFALKVIYGNHEDTVRRQICREIEILRSVDHPNVVKCHDMFDHNGEIQVLLEFMDQGSLEGAHIWQEQELADLSRQILSGLAYLHRRHIVHRDIKPSNLLINSAKNVKIADFGVSRILAQDMDPCNDSVGTIAYMSPERFDSAAGENSDVYAGDVWSLGVSILEFYLGRFPFAVSRQGDWASLMCAICMSQPPEAPATASQEFRHFVSCCLQSDPPKRWSAQQLLQHPFILKATGGPNLRQMLPPPRPLPSAS...(TRANSITION TO YFP)

>MKK5^DD^-7B

MKPIQSPSGVASPMKNRLRKRPDLSLPLPHRDVALAVPLPLPPPSSSSSAPASSSAISTNISAAKSLSELERVNRIGSGAGGTVYKVIHTPTSRPFALKVIYGNHEDTVRRQICREIEILRSVDHPNVVKCHDMFDHNGEIQVLLEFMDQGSLEGAHIWQEQELADLSRQILSGLAYLHRRHIVHRDIKPSNLLINSAKNVKIADFGVSRILAQDMDPCNDSVGTIAYMSPERINTDLNHGRYDGYAGDVWSLGVSILEFYLGRFPLLPQGQRPDWASLMCAICMSQPPEAPATASQEFRHFVSCCLQSDPPKRWSAQQLLQHPFILKATGGPNLRQMLPPPRPLPSAS...(TRANSITION TO YFP)

>N7-MKK5^DD^-C7

MALVRKRRQINLRLPVPPLSVHLPWFSFASSTAPVINNGISASDVEKLNRIGSGAGGTVYKVIHTPTSRPFALKVIYGNHEDTVRRQICREIEILRSVDHPNVVKCHDMFDHNGEIQVLLEFMDQGSLEGAHIWQEQELADLSRQILSGLAYLHRRHIVHRDIKPSNLLINSAKNVKIADFGVSRILAQDMDPCNDSVGTIAYMSPERFDSAAGENSDVYAGDIWSFGVMILELFVGHFPLLPQGQRPDWATLMCVVCFGEPPRAPEGCSDEFRSFVDCCLRKESSERWTASQLLGHPFLRESL...(TRANSITION TO YFP)

>N5-MKK7^ED^-C5

MKPIQSPSGVASPMKNRLRKRPDLSLPLPHRDVALAVPLPLPPPSSSSSAPASSSAISTNISAAKSLSELERVHVLGRGSSGIVYKVHHKTTGEIYALKSVNGDMSPAFTRQLAREMEILRRTDSPYVVRCQGIFEKPIVGEVSILMEYMDGGNLESLRGAVTEKQLAGFSRQILKGLSYLHSLKIVHRDIKPANLLLNSRNEVKIADFGVSKIITRELDYCNDYVGTCAYMSPERINTDLNHGRYDGYAGDVWSLGVSILEFYLGRFPFAVSRQGDWASLMCAICMSQPPEAPATASQEFRHFVSCCLQSDPPKRWSAQQLLQHPFILKATGGPNLRQMLPPPRPLPSAS...(TRANSITION TO YFP)

>N7-MKK6^EE^-C7

MALVRKRRQINLRLPVPPLSVHLPWFSFASSTAPVINNGISASDVEKLKVIGKGSGGVVQLVRHKWVGKFFAMKVIQMNIQEEIRKQIVQELKINQASSQCPHVVVCYHSFYHNGAFSLVLEYMDRGSLADVIRQVKTILEPYLAVVCKQVLLGLVYLHNERHVIHRDIKPSNLLVNHKGEVKISDFGVSASLASEMGQRDEFVGTYNYMSPERFDSAAGENSDVYAGDIWSFGVMILELFVGHFPLLPQGQRPDWATLMCVVCFGEPPRAPEGCSDEFRSFVDCCLRKESSERWTASQLLGHPFLRESL...(TRANSITION TO YFP)
